# Evaluating the Efficacy and Safety of the Thumper Device for Cardiac Arrest: A Systematic Literature Review and Meta-Analysis

**DOI:** 10.31083/j.rcm2407191

**Published:** 2023-07-03

**Authors:** Ding Luo, Yuji Weng, Na Zhang, Baichao Xu, Hua Zhang, Jiameng Wang

**Affiliations:** ^1^International Nursing School of Hainan Medical University, 571199 Haikou, Hainan, China; ^2^Key Laboratory of Emergency and Trauma, Ministry of Education, 571199 Haikou, Hainan, China; ^3^Research Unit of Island Emergency Medicine, Chinese Academy of Medical Sciences, 571199 Haikou, Hainan, China; ^4^The Second Affiliated Hospital of Hainan Medical University, 571199 Haikou, Hainan, China; ^5^Department of Physical Education Hainan Medical University, 571199 Haikou, Hainan, China; ^6^Faculty of Physical Education, Yan’an University, 716000 Yan’an, Shaanxi, China

**Keywords:** cardiac arrest, cardiopulmonary resuscitation (CPR), thumper, manual chest compression, meta-analysis, emergency care

## Abstract

**Background:**

Cardiopulmonary resuscitation (CPR) is a major rescue measure for cardiac arrest (CA) patients, and chest compression is the key to CPR. The Thumper device was designed to facilitate manual compression during CPR. However, current randomized controlled trials (RCTs) provide controversial findings on the efficacy of the Thumper device.

**Objectives:**

This meta-analysis aimed to compare the clinical benefits of using the Thumper device with manual chest compressions during the provision of CPR for patients in CA.

**Methods:**

Relevant studies were retrieved from various databases, including Ovid, PubMed, Web of Science, EMBASE, Cochrane, and CNKI, and by manually searching the reference lists of research and review articles. All RCTs published in either English or Chinese until June 31, 2020, were included in the meta-analysis. The odds ratios (ORs) and their 95% confidence intervals (95% CIs) for the return of spontaneous circulation (ROSC), survival rate (SR), and the incidence of rib fractures (RFs) were compared between the manual and Thumper chest compressions.

**Results:**

A total of 2164 records were identified, of which 16 were RCTs with an overall risk of bias ranging from low to medium classification. Following CPR, the odds ratios for ROSC, SR, and RF were significantly better for the Thumper chest compression with ORs of 2.56 (95% CI 2.11–3.11, $I^{2}$ = 0%), 4.06 (95% CI 2.77–5.93, $I^{2}$ = 0%), and 0.24 (95% CI 0.14–0.41, $I^{2}$ = 0%), respectively.

**Conclusions:**

The Thumper compression devices may improve patient outcome, when used at inhospital cardiac arrest. This review suggests a potential role for mechanical chest compression devices for in-hospital cardiac arrest, but there is an urgent need for high-quality research, particularly adequately powered randomised trials, to further examine this role.

## 1. Introduction

Cardiac arrest (CA) is a medical emergency caused by the abrupt loss of heart function resulting in a sudden loss of blood flow [1]. CA is the leading cause of death worldwide. In the United States and Europe, more than 300,000 and 450,000 people, respectively, die of CA each year [2, 3]. The performance of timely cardiopulmonary resuscitation (CPR) determines the survival rate as well as the neurological outcome in patients suffering from CA [4, 5]. The quality of the chest compression is essential during CPR to maintain organ perfusion which ultimately determines the prognosis of CA patients.

Chest compressions can be provided manually or mechanically. The main advantage of manual chest compression is that it can be administered immediately at the scene and, hence, improves the chances of survival [5]. However, the CPR procedure is also physically demanding. During prolonged CPR, the rescuer’s fatigue can reduce the quality of the chest compressions, particularly if the procedure is performed outside the hospital or on hospital transport [6].

In order to overcome the limitations of manual chest compressions, several mechanical compression devices have been proposed. Namely point chest compression, suction chest compression, full chest coverage load distribution compression (vest type), and broad chest compression (three-dimensional type) [7]. Some experimental studies have shown that mechanical chest compression can provide more uniform recoil and increase the intrathoracic pressure when compared with manual chest compression, thus increasing the effective coronary perfusion pressure and systemic blood flow [7, 8, 9]. This provides an advantage when the CPR is performed outside the hospital or on ambulances [10]. The Thumper compression device uses point chest compression and is seeing an increase in use in the rescue of CA patients. When using a Thumper device, the rescuers can focus on providing supplementary advanced life support, as they no longer need to worry about providing manual chest compressions. This may improve the efficiency and outcomes of the rescue operation. Additionally, compared with manual compression, the Thumper device provides constant high-quality chest compression, frequency, depth and rhythm. This eliminates the inconsistencies caused by operator fatigue following a prolonged manual resuscitation and changing operators throughout the procedure.

However, the clinical benefit of using the Thumper device as opposed to the manual compression is still not clear. There is an obvious inconsistency in the published literature on the efficacy of Thumper compression. Various randomized controlled trials (RCTs) can not demonstrate that Thumper compression can improve survival when compared with manual compression. Nevertheless, several observational studies showed that Thumper device compression could improve the survival rate [11, 12]. In addition, there is a lack of data on the safety profile of the Thumper device. Therefore there is a need for a meta-analysis to summarize the relative effectiveness and safety of Thumper devices in relation to manual compression in patients with CA. In view of this, we conducted a meta-analysis to comprehensively evaluate the effects of Thumper compression and manual compression in patients with cardiac arrest.

## 2. Materials and Methods

The meta-analysis was conducted in accordance with the agreement registered in the PROSPERO database on September 25, 2020 (Registration Number: CRD420206025).

### 2.1 Information Sources

Relevant studies published before June 30, 2020, were retrieved from the Ovid, PubMed, Web of Science, EMBASE, Cochrane Library, Trials registries, Google Scholar, and the China National Knowledge Infrastructure (CNKI) electronic databases. In addition, snowballing was used to identify relevant research articles from the reference list of published studies. The Google Academy was used to identify and screen studies that cited such evidence. On April 28, 2020, we conducted a search on Google Academy and conducted a supplementary search on the websites of relevant organizations, including government departments and research institutions. The database search was again updated on November 15, 2022, and a final literature and snowball search was performed on November 22, 2022.

### 2.2 Search Strategy

Twelve known related studies [1, 11, 12, 13, 14, 15, 16, 17, 18, 19, 20, 21] were used to identify records in the electronic databases. We determined the candidate search terms by screening the records’ titles, abstracts, and search processes. These terms were then used to formulate a draft of the search strategy, and other search terms were determined according to the results of the strategy. The PubReMiner tool was used to identify and check the frequency of the search terms. The MEDLINE policy uses the Cochrane RCT filter reported in the Cochrane manual version 5.2. The search strategy was limited to only English or Chinese language articles and there was no restriction on publication. The search strategy was then verified by ensuring that it could identify the 12 known related studies on the PubMed and EMBASE databases. The search strategy was developed by experienced researchers among the project members. The overall structure and accuracy of the final search strategy were discussed and peer-reviewed by Zhang. The final search strategy used a combination of keywords to describe the condition (cardiac arrest), intervention (compression device), and study design (RCT).

### 2.3 Inclusion and Exclusion Criteria

All RCTs on adult (age ≥18 years) CA patients published in either English or Chinese that compared clinical outcomes between the Thumper and manual chest compressions were included in the meta-analysis. The primary clinical outcome measure for this meta-analysis was the return of spontaneous circulation (ROSC), and the secondary outcome measures were discharge survival rate and the incidence of rib fractures. If these all of the outcomes indicators were not published in the study, the corresponding author of the research articles was contacted via e-mail to provide the additional data. If these data were not provided, the article was excluded.

All studies using mechanical chest compression with mechanical devices other than the Thumper were excluded. Additionally, studies that included children under 18 years, animal and simulation studies, non-RCTs, and those lacking controls were excluded. Unpublished manuscripts and conference abstracts were also excluded.

### 2.4 Selection Process

Two researchers (Luo and Zhang) independently reviewed the titles and abstracts of all records. The researchers then filtered the titles and abstracts of all retrieved articles to identify the full-text articles for inclusion. If the included articles met the eligibility criteria, the full text was further searched to ensure that all required data were available. The references of the articles were also reviewed to identify additional suitable studies. Any disagreements in the retrieved articles were resolved through discussion. If the researchers failed to reach an agreement, a third researcher (Wang) was consulted for the final decision.

### 2.5 Data Collection Process

The general information of the research article (first author name, year of study, and publication date), the sample size of the control group and experimental group, and the type of study were extracted from the articles. Additionally, the American Heart Association (AHA) guidelines used to define the control group, the intervention measures of the experimental and control groups, and the outcome indicators ROSC and discharge survival and the incidence of rib fractures were also extracted. All the data were extracted by three researchers (Luo, Zhang, and Wang) and recorded on an excel sheet. The extracted data were compared, and any differences were resolved through discussion. Finally, one of the researchers (Luo) entered the data into the Review Manager (ReMan) 5.3 software (Nordic Cochrane Center, London, UK) and checked their accuracy. If any part of the above data were not clearly described in the research article, the corresponding author was consulted to provide further details.

### 2.6 Risk of Bias Assessment

The included studies were assessed for bias in accordance with the evaluation of the authenticity of RCT criteria published by the Cochrane Collaboration Network. This assessment is based on seven criteria: random sequence generation, allocation concealment, blinding of participants and personnel, blinding of outcome assessment, incomplete outcome data, selective reporting, and other biases. Two reviewers (Luo and Zhang) independently applied the risk assessment tools to all included studies and classified the risk bias of each study as low-risk, high-risk, or unclear. A justification for the classification was also provided. Any differences in determining the risk of bias or justification were resolved through discussions between the two reviewers (Luo and Zhang). If necessary, a third reviewer (Wang) acted as an arbitrator.

Funnel plot analysis was used to estimate the publication bias. If the funnel plot was asymmetric, the research articles were reviewed to identify possible sources of bias, such as publication or trial design bias. A sensitivity analysis was performed to determine the robustness of the observed outcomes.

### 2.7 Statistical Analysis

The data were processed using the Revman 5.3 developed by the Cochrane Collaboration. The odds ratios (OR) or mean differences (MDs) were reported for dichotomous and continuous variables. The I-squared ($I^{2}$) test was used to assess the heterogeneity of the included studies. An $I^{2}$ greater or equal to 50% indicates high heterogeneity between studies [22]. If the statistical heterogeneity among studies was high, the subgroup analysis was deemed invalid, and the random effect model (RE) was used to analyze the statistical indicators. Conversely, the fixed effect (FE) model was used to analyze the statistical indicators if the statistical heterogeneity was low. The differences in clinical outcomes between the two compression methods were deemed statistically significant if the *p*-value was below 0.05.

### 2.8 Ethical Considerations

This study was conducted in compliance with the recommendations published by the preferred reporting item of the guidelines (PRISMA 2020) for meta-analysis and systematic reviews. Since no patient data were collected in this study, ethical approval was not required.

## 3. Results

### 3.1 Searching Results/Study Selection

**Fig. 1.**
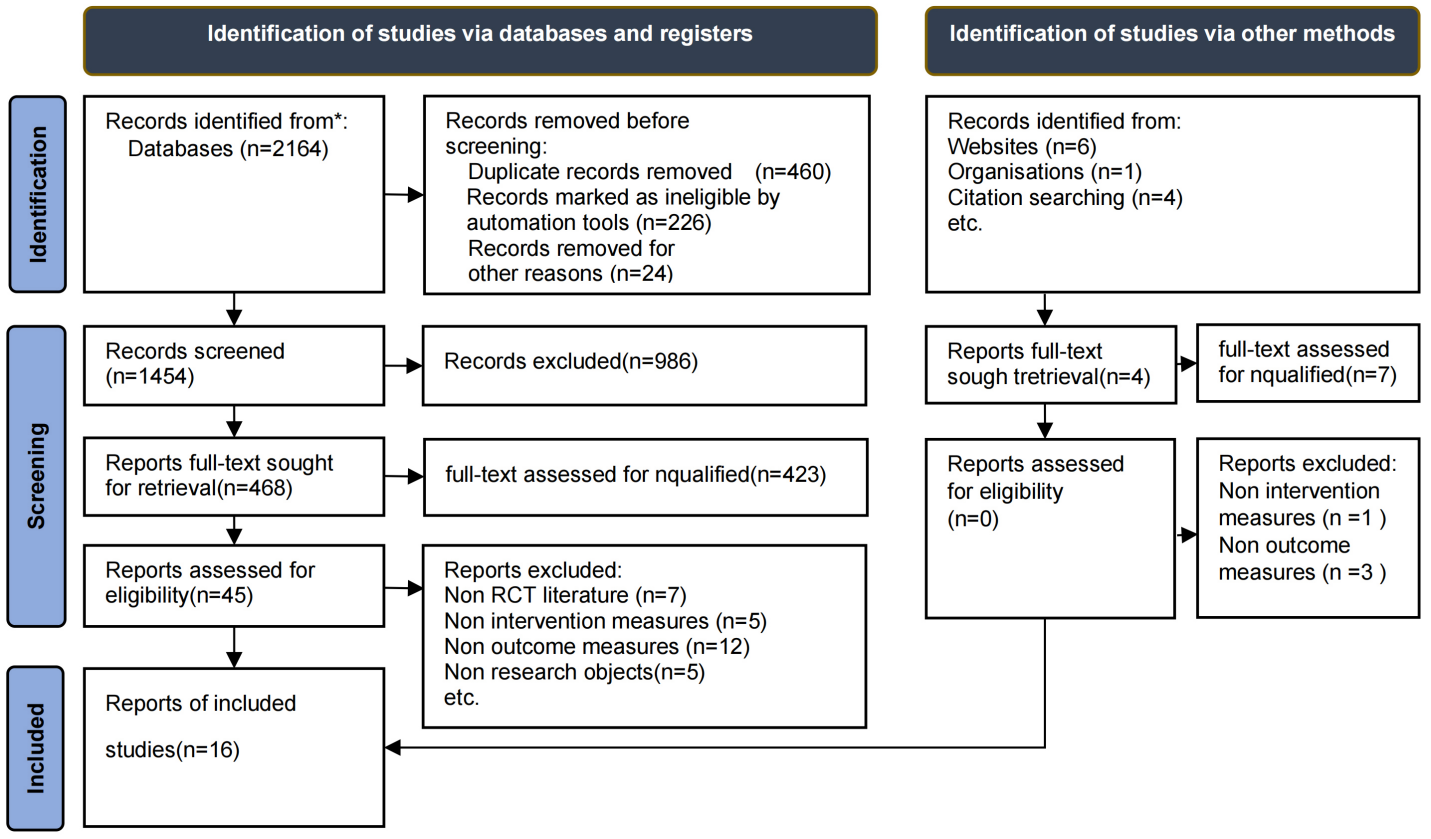
**2020 PRISMA flowchart of the literature search and study selection process for new systematic reviews, which included searches of databases, registers, and other sources**. RCT, randomized controlled trials.

The studies identified during the literature screening process are summarized in Fig. 1. A total of 2164 records were retrieved in our database search, of which 11 articles were obtained by snowballing. After removing the duplicate items, we identified 1454 relevant records, of which only 45 were full-text articles. From these full-text articles, only 16 papers met the eligibility criteria stated above. Later, we searched for all references that ultimately included evidence. However, no other articles identified through this search met the eligibility criteria.

### 3.2 Study Characteristics and Quality

**Fig. 2.**
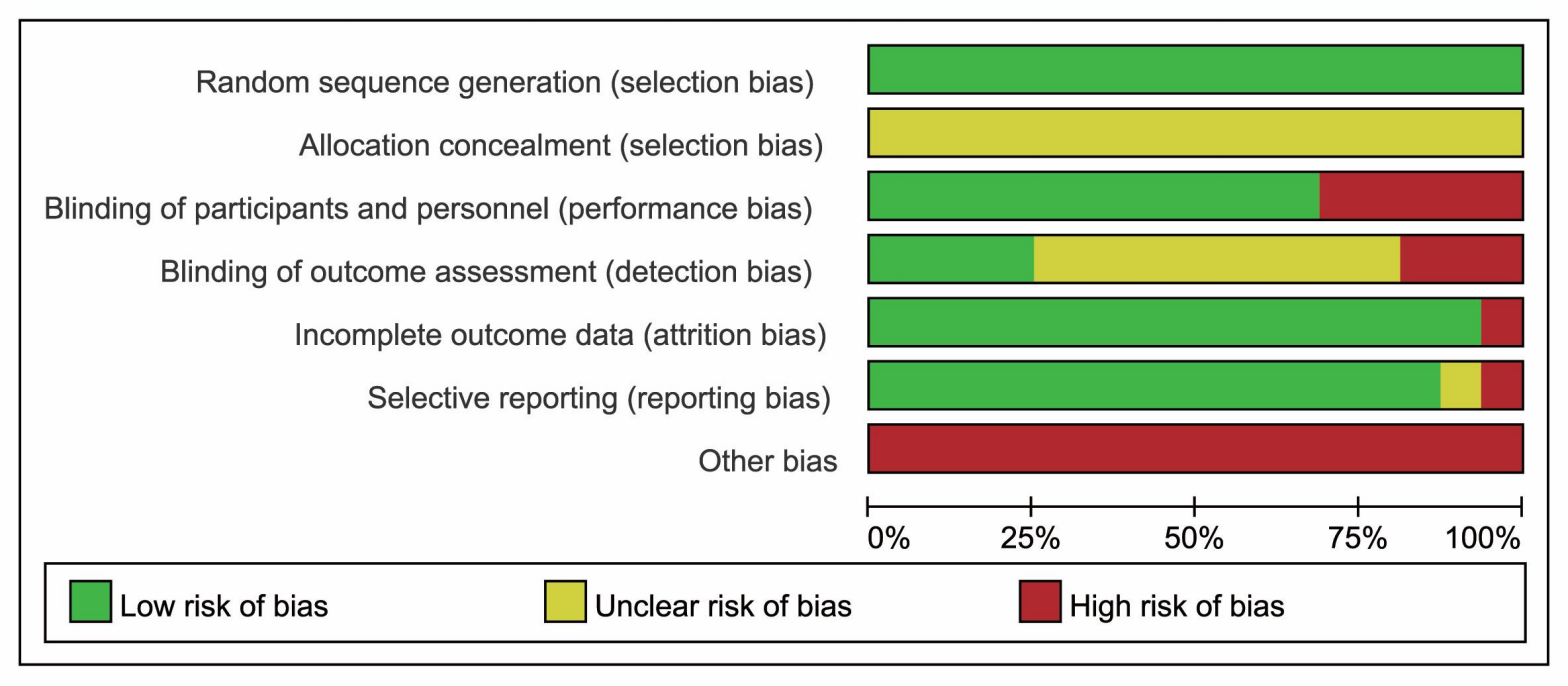
**Risk of bias summary for included studies**.

This meta-analysis included 16 RCTs involving 2275 CA patients. The characteristics of these studies and the participants are summarized in Table 1 (Ref. [23, 24, 25, 26, 27, 28, 29, 30, 31, 32, 33, 34, 35, 36, 37, 38]). All 16 articles used the random grouping method, and 4 of them [23, 24, 25, 26] reported the exact random method used. The Cochrane bias risk assessment tool was used for quality assessment and bias risk assessment was done at study level, all 16 articles had an overall risk of bias, ranging from low to high, as shown in Table 2 (Ref. [23, 24, 25, 26, 27, 28, 29, 30, 31, 32, 33, 34, 35, 36, 37, 38]) and Fig. 2.

**Table 1.** **Characteristics of the included trials and participants**.

Author and year	Country	Setting	Year of study	Sample (T/C)	Study design	Age (T/C)	Men (%)	AHA version	ROSC (T/C)	Discharge Survival rate (T/C)	Rib fracture (T/C)
Meng LF 2019 [23]	China	ER	2015–2018	200 (100/100)	RCT	(41.03 ± 3.11)/ (42.01 ± 2.17)	56.5	2005	76/46	59/24	NR
Zheng H 2019 [27]	China	ER	2018–2018	100 (48/52)	RCT	61	61	2015, 2010	27/18	NR	NR
Zhang CY 2017 [28]	China	ER	2015–2017	150 (70/80)	RCT	(59.18 ± 11.98)/ (62.65 ± 8.57)	78.7	2010	48/41	NR	NR
You Y 2017 [29]	China	ER	2015–2016	80 (40/40)	RCT	(63.71 ± 13.97)/ (67.74 ± 15.21)	60	2015	32/20	NR	0/4
He NN 2016 [24]	China	IHCA	2005–2011	400 (200/200)	RCT	43.2 ± 5.33	72	2005, 2010	166/131	NR	NR
Gong N 2016 [25]	China	ER	2010–2014	247 (112/135)	RCT	(61 ± 17)/ (61 ± 18)	62.3	2010	Not applicable	18/4	NR
Dong QL 2016 [30]	China	IHCA	2012–2015	100 (50/50)	RCT	(64.12 ± 5.07)/ (63.71 ± 4.23)	56	2005	46/28	NR	0/6
Liu HL 2015 [31]	China	IHCA	2012–2014	135 (65/70)	RCT	(68.37 ± 11.16)/ (67.99 ± 11.09)	59.2	2010	42/35	NR	2/9
Ding HB 2014 [26]	China	ER	2011–2014	68 (34/34)	RCT	(45~65)/ (46~68)	54.4	2005	23/13	16/6	0/5
Guo S 2014 [32]	China	IHCA	2009–2012	158 (80/78)	RCT	(51.23 ± 9.86)/ (50.30 ± 10.18)	58.9	2010	43/24	NR	1/4
Jin Y 2013 [33]	China	IHCA	2008–2012	146 (71/76)	RCT	14~75	47.8	2008	31/21	NR	NR
Liu JF 2013 [34]	China	IHCA	2013	32 (16/16)	RCT	39~75	59.3	2010	5/3	NR	NR
Hu PB 2012 [35]	China	ER	2010–2012	107 (55/52)	RCT	(47.38 ± 13.35)/ (45.44 ± 12.43)	52.3	2005	30/18	NR	7/20
Huang Q 2011 [36]	China	IHCA	2008–2009	152 (62/91)	RCT	19~76	63.1	2005	25/12	10/4	NR
Lu XG 2010 [37]	China	ER	2009–2010	150 (74/76)	RCT	(47.72 ± 14.25)/ (45.50 ± 13.82)	59.3	2005	42/28	25/11	2/8
Taylor 1978 [38]	America	ER	—–	50 (26/24)	RCT	—	—	—	10/10	3/2	7/8

NR, not reported; RCT, randomized controlled trial; ROSC, return of spontaneous circulation; T/C, Thumper group/Control group; ER, emergency room; IHCA, in-hospital cardiac arrest; AHA, American Heart Association.

**Table 2.** **Evaluation of risk assessment of the included RCT according to the Cochrane Collaboration Network**.

Author and year	1. Random sequence generation	2. Allocation concealment	3. Blinding of participants and personnel	4. Blinding of outcome assessment	5. Incomplete outcome data	6. Selective reporting	7. Other bias
Meng LF 2019 [23]	Low risk	Unclear risk	Low risk	unclear	Low risk	Low risk	High risk
Zheng H 2019 [27]	Low risk	Unclear risk	High risk	unclear	Low risk	Low risk	High risk
Zhang CY 2017 [28]	Low risk	Unclear risk	Low risk	Low risk	Low risk	Low risk	High risk
You Y 2017 [29]	Low risk	Unclear risk	High risk	unclear	Low risk	Low risk	High risk
He NN 2016 [24]	Low risk	Unclear risk	Low risk	unclear	Low risk	Low risk	High risk
Gong N 2016 [25]	Low risk	Unclear risk	Low risk	Low risk	Low risk	Low risk	High risk
Dong QL 2016 [30]	Low risk	Unclear risk	Low risk	unclear	Low risk	Low risk	High risk
Liu HL 2015 [31]	Low risk	Unclear risk	Low risk	unclear	Low risk	Low risk	High risk
Ding HB 2014 [26]	Low risk	Unclear risk	Low risk	Low risk	Low risk	Low risk	High risk
Guo S 2014 [32]	Low risk	Unclear risk	High risk	unclear	Low risk	High risk	High risk
Jin Y 2013 [33]	Low risk	Unclear risk	High risk	High risk	Low risk	Low risk	High risk
Liu JF 2013 [34]	Low risk	Unclear risk	Low risk	Low risk	Low risk	Low risk	High risk
Hu PB 2012 [35]	Low risk	Unclear risk	Low risk	High risk	Low risk	Low risk	High risk
Huang Q 2011 [36]	Low risk	Unclear risk	Low risk	unclear	Low risk	Low risk	High risk
Lu XG 2010 [37]	Low risk	Unclear risk	Low risk	High risk	Low risk	Low risk	High risk
Taylor 1978 [38]	Low risk	Unclear risk	High risk	unclear	High risk	unclear	High risk

RCT, randomized controlled trial.

### 3.3 ROSC Rate

**Fig. 3.**
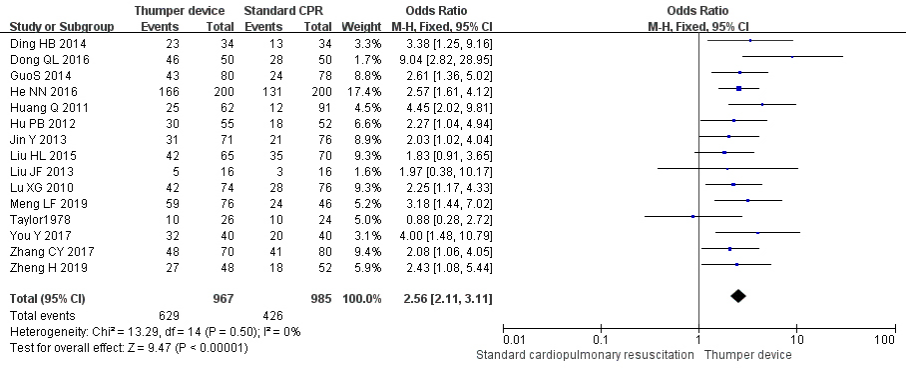
**The ROSC forest plot of Thumper chest compression versus manual chest compression**. CPR, cardiopulmonary resuscitation; CI, confidence interval; ROSC, return of spontaneous circulation.

Among the 16 RCT studies, 15 studies [23, 24, 26, 27, 28, 29, 30, 31, 32, 33, 34, 35, 36, 37, 38] compared the ROSC between the mechanical and manual chest compressions. Thirteen of these studies [23, 24, 26, 27, 28, 29, 30, 31, 32, 33, 35, 36, 37] showed that ROSC in the Thumper chest compression group was significantly higher when compared with the manual chest compression group (*p* < 0.05). In contrast, no significant difference was reported in the other two studies conducted by Taylor *et al*. [38] and Liu *et al*. [34]. The meta-analysis showed that the ROSC in the Thumper chest compression group was better than that in the manual chest compression group (OR = 2.56, 95% CI 2.11~3.11, Z = 9.47, *p* < 0.05). The forest plots of this analysis are presented in Fig. 3.

### 3.4 Discharge Survival Rate

**Fig. 4.**
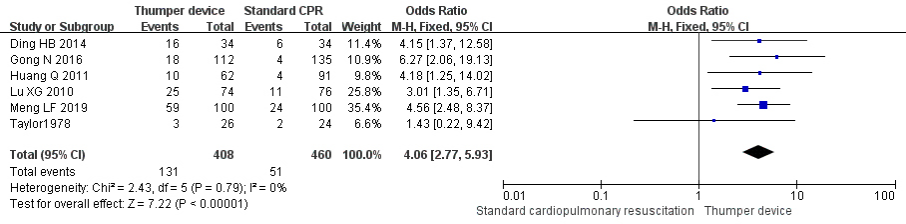
**The discharge survival rate forest plot of Thumper chest compression versus manual chest compression**. CPR, cardiopulmonary resuscitation; CI, confidence interval.

Among the 16 RCT studies, six studies compared [23, 25, 26, 36, 37, 38] the discharge survival rate. Five of these studies [23, 25, 26, 36, 37] showed that the discharge survival rate in the Thumper chest compression group was significantly higher (*p* < 0.05) than that of the manual chest compression group, while no significant difference (*p >* 0.05) was noted in the study by Taylor *et al*. [38]. The meta-analysis results showed that the discharge survival rate in the Thumper chest compression group was significantly higher when compared with the manual chest compression group (OR = 4.06, 95% CI 2.77–5.93, Z = 7.22, *p* < 0.05). The forest plot analysis is presented in Fig. 4.

### 3.5 Incidence of Rib Fractures 

**Fig. 5.**
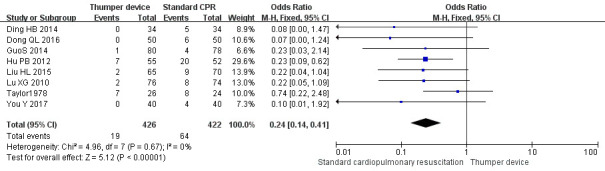
**Incidence of rib fractures forest plot of Thumper chest compression versus manual chest compression**. CPR, cardiopulmonary resuscitation; CI, confidence interval.

Among the 16 RCTs included, eight studies [26, 29, 30, 31, 32, 35, 37, 38] compared the incidence of rib fractures. Seven of these studies [26, 29, 30, 31, 32, 35, 37] showed that the incidence of rib fractures in the Thumper chest compression group was significantly lower (*p* < 0.05) when compared with the manual chest compression group. There was no heterogeneity among the studies (*p* = 0.67, $I^{2}$ = 0%, $I^{2}$ <50%), so the FE model was used for meta-analysis. The meta-analysis results showed that the incidence of rib fractures in the Thumper chest compression group was significantly lower than that of the manual chest compression group (OR = 0.24, 95% CI 0.14–0.41, Z = 5.12, *p* < 0.05). The forest plot analysis is illustrated in Fig. 5.

### 3.6 Publication Bias

**Fig. 6.**
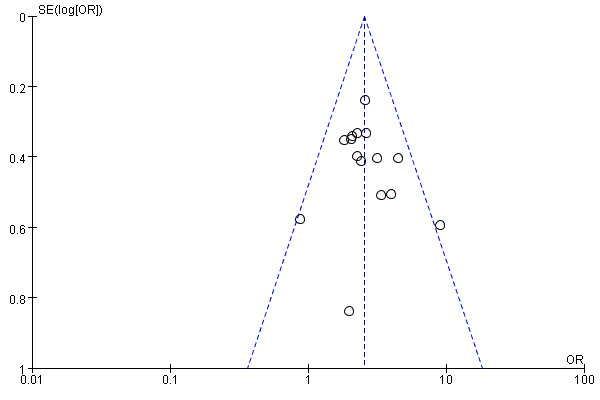
**Funnel plot of the included paper**. OR, odds ratio; SE, standard error.

The ROSC funnel plot shape was inverted and symmetrical, indicating no significant bias (Fig. 6). The symmetry of the survival rate and rib fracture funnel plots could not be evaluated due to limited studies evaluating these outcomes. However, when considering the small sample size in these studies, publication bias could not be ruled out.

### 3.7 Sensitivity Analysis

**Fig. 7.**
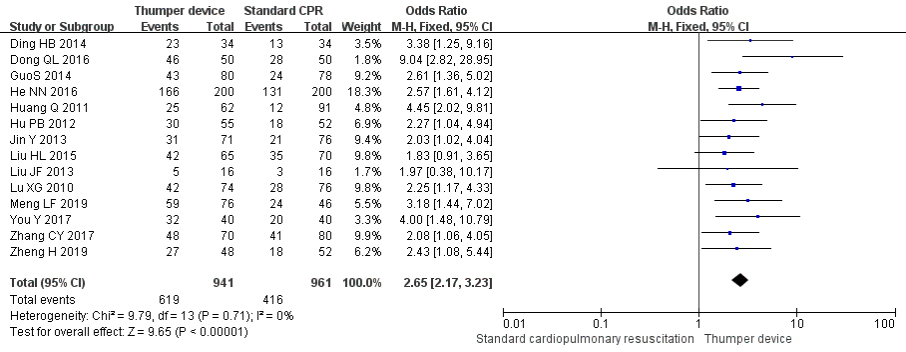
**The ROSC Subgroup analysis forest plot of Thumper device group versus manual CPR group**. CPR, cardiopulmonary resuscitation; CI, confidence interval; ROSC, return of spontaneous circulation.

**Fig. 8.**
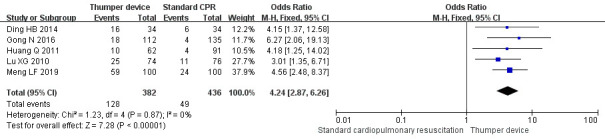
**The survival rate Subgroup analysis forest plot of Thumper device group versus manual CPR group**. CPR, cardiopulmonary resuscitation; CI, confidence interval.

**Fig. 9.**
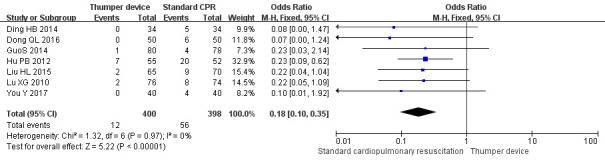
**The rib fracture rate Subgroup analysis forest plot of Thumper device group versus manual CPR group**. CPR, cardiopulmonary resuscitation; CI, confidence interval.

The sensitivity analysis forest plots are shown in Figs. 7,8,9. The sensitivity analysis showed that the difference between before and after each effect index was small. The difference between the relative risk (RR) and OR models was not obvious. The outcomes of the study of None *et al*. [38] varied when compared with the other studies, in order to exclude bias caused by language differences and it was therefore excluded from the sensitivity analysis. The results of the effect indicators for the ROSC, survival rate, and incidence of rib fractures were 2.65 (95% CI 2.17–3.23, Z = 9.65), 4.24 (95% CI 2.87–6.26, Z = 7.28), and 0.18, (95% CI 0.10–0.35, Z = 5.22), respectively. For all outcome indicators, the results were statistically significant (*p* < 0.00001), indicating that the findings of the meta-analysis are highly reliable.

## 4. Discussion

The application of CPR for CA patients aims to restore spontaneous circulation as soon as possible and to obtain better neurological function after discharge. The 2015 AHA guidelines for CPR emphasize that early high-quality CPR is crucial to improving survival and neurological function in CA patients [6]. Current methods of compression include manual compression and mechanical compression, each of which has advantages and disadvantages. However, current study provide controversial findings. Couper *et al*. [11] showed that chest compression provided a better 30-day survival rate and short-term survival rate when compared with manual chest compression. In addition, Westfall *et al*. [21] showed that mechanical chest compression could significantly improve ROSC and load compression distribution compared with manual compression. However, only a few study showed that mechanical chest compression could significantly improve the prognosis of CA patients [11, 21]. In contrast, several study conducted in recent years with more participants concluded that the use of mechanical compression devices did not improve prognosis compared with manual chest compressions [1, 15]. In a study performed by Zhu *et al*. [14], the ROSC rate, in-hospital survival rate, discharge survival rate, and CPC score in the manual chest compression group were significantly better than those of the mechanical chest compression group. The study conducted by Khan *et al*. [15] showed that mechanical chest compression performed resulted in a similar 30-day survival rate, discharge survival rate, admission survival rate, ROSC, neurological function recovery, and rib fracture incidence when compared with manual chest compressions. However, although some studies concluded that mechanical compression has no obvious advantage in the prognosis of CA patients, it can be difficult to provide high-quality chest compression in some special circumstances, such as during transportation, operating conditions, and in situations whereby the safety of the rescuers is at risk. Therefore the use of mechanical chest compression is still high.

To our knowledge, there is currently no meta-analysis comparing the survival and prognosis between the Thumper and manual chest compressions.

A total of 16 RCTs were included in this meta-analysis. The results showed that there were differences between manual compression and mechanical compression in terms of the three observed indicators (the ROSC rate, the rate of survival to hospital discharge, and Incidence of rib fractures). There were several methodological differences between studies that could have influenced the findings of our meta-analysis, such as the number of times, duration, and locations of the CPR. Furthermore, mechanical chest compression devices and CPR practices are constantly improving, potentially explaining the worse outcomes noted in earlier studies such as the one by Taylor *et al*. [38].

The results of this meta-analysis differ from the findings of other published meta-analyses. Liu *et al*. [1] evaluated the data of six studies with a total of 8501 participants, and found that the use of LUCAS chest compression devices did not improve ROSC (OR = 1; 95% CI: [0.89, 1.13]) and hospital survival (OR = 0.86; 95% CI: [0.65, 1.15]). Bonnes *et al*. [17] conducted a large-sample review (n = 9157) of data from observational studies. They concluded that the use of mechanical devices could significantly improve short-term outcomes such as ROSC and discharge survival. Still, no significant benefit was observed in the long-term prognosis after discharge.

Three reasons could explain the different findings of our meta-analysis. In previous meta-analyses, chest compression devices with a three-dimensional compression mode were used as opposed to the single-point compression mode used by the Thumper device. The Cochrane quality assessment classified the studies as very low in quality or uncertain. Meta-analyses based on low-quality studies may overestimate or underestimate the effectiveness of treatment [39]. Therefore the current evidence is not sufficient to support the effect of the Thumper device of resuscitation, particularly for the assessment of survival and incidence of rib fractures. Finally, differences in the operating environment could lead to differences in resuscitation outcomes. Spiro *et al*. [40] and Parnia *et al*. [41] have shown that mechanical devices are more effective in resuscitation and maintaining patients’ survival than manual chest compressions for CA patients in the hospital environment. This may be related to the ability to provide greater team support in-hospital than out-of-hospital after cardiac arrest. Moreover, mechanical chest compression devices can be deployed earlier in the hospital environment, potentially improving outcomes. However, the meta-analysis of Bonnes *et al*. [17] has shown that the earlier the mechanical device is deployed during a CA in the out-of-hospital environment, the more effective the CPR is. It is important to note that chest compression pauses related to the deployment of a mechanical chest compression device are rarely reported. Studies have shown that the rescuer’s skills can have an influence on the deployment speed and performance of mechanical chest compression, which could also have an impact on outcomes [11, 42]. In addition, in the hospital environment, manual chest compression is usually difficult to implement because patients are usually positioned on a compressible mattress, which can absorb up to 40% of the compression force, resulting in a lower compression depth than the standard required by the AHA guidelines [43].

The most important strength of this system review and meta analysis is the first analysis of the survival of Thumper mechanical chest compression. This is of clinical relevance with regard to increasing treatment options in hospital, where automated mechanical CPR devices might provide a “bridge” to definitive treatment for designated patient groups under certain conditions and in specific environments. We hope that this systematic review and meta- analysis may contribute to implicate future clinical and scientific issues towards an individualised decision making.

This meta-analysis has some limitations that have to be acknowledged. The overall Cochrane quality evaluation showed that the quality of the RCTs evaluated in the study was often low. Not all RCTs evaluated in this study explained the technique used to randomize the participants into the mechanical and manual compression groups and the blinding method used to evaluate clinical outcomes. Details on the patients lost to follow-up were not always provided. The limited number of studies included in this meta-analysis and the small sample size of the included studies may limit the generalizability of the research findings. Therefore further high-quality RCTs are recommended to confirm the effect of the Thumper device. In addition, this study only evaluated the outcomes of the Thumper chest compression in relation to manual chest compression in CA patients, highlighting the need for further research to evaluate the outcomes of other mechanical chest compression devices. It is also important to note that long-term survival is affected by many factors, such as the severity of CA, patient co-morbidities, and follow-up treatment. Therefore these findings may not reflect the long-term survival outcomes, highlighting the need for further longitudinal studies. Finally, this meta-analysis did not evaluate the clinical impact of other co-founding variables such as etiology and location of the CA procedure (e.g., within a hospital or outside the hospital), and hence more research is required to assess the impact of these variables on clinical outcomes to better guide rescuers on the use of mechanical chest compression in CA patients.

## 5. Conclusions

In this review, our meta-analysis found an association between improved hospital survival and treatment with a Thumper compression device for in-hospital cardiac arrest. We also found evidence of improved short-term survival and improved physiological outcomes when a mechanical device was used. Nevertheless, it is important to note that the Thumper device may not always be available to the rescuers. This review suggests a potential role for mechanical chest compression devices for in-hospital cardiac arrest, but there is an urgent need for high-quality research, particularly adequately powered randomised trials, to further examine this role.

## Supplementary Material

Supplementary material associated with this article can be found, in the online version, at https://doi.org/10.31083/j.rcm2407191.
